# Supplementary material for: Modeling glioblastoma heterogeneity as a dynamic network of cell states
Source: Mol Syst Biol. 2021 Sep 16;17(9):e10105. doi: 10.15252/msb.202010105 (PMC8444284; doi:10.15252/msb.202010105)
Supplement: Supplementary file 6 — Source Data for Figure 5 [file MSB-17-e10105-s004.zip › Figure5A_sourcedata/GSEA_3017/hallmarks_stateC.GseaPreranked.1621934520804/HALLMARK_EPITHELIAL_MESENCHYMAL_TRANSITION.html]

Details for gene set HALLMARK\_EPITHELIAL\_MESENCHYMAL\_TRANSITION[GSEA]

|  || Dataset | state13017 |
| Phenotype | NoPhenotypeAvailable |
| Upregulated in class | na\_neg |
| GeneSet | HALLMARK\_EPITHELIAL\_MESENCHYMAL\_TRANSITION |
| Enrichment Score (ES) | -0.46240675 |
| Normalized Enrichment Score (NES) | -2.4451954 |
| Nominal p-value | 0.0015197569 |
| FDR q-value | 6.071429E-4 |
| FWER p-Value | 0.001 |
Table: GSEA Results Summary

  

Fig 1: Enrichment plot: HALLMARK\_EPITHELIAL\_MESENCHYMAL\_TRANSITION      
 Profile of the Running ES Score & Positions of GeneSet Members on the Rank Ordered List

  

| PROBE | GENE SYMBOL | GENE\_TITLE | RANK IN GENE LIST | RANK METRIC SCORE | RUNNING ES | CORE ENRICHMENT || 1 | GAS1 |  |  | 64 | 0.384 | -0.0905 | No |
| 2 | MCM7 |  |  | 91 | 0.352 | -0.1150 | No |
| 3 | DPYSL3 |  |  | 95 | 0.346 | -0.0987 | No |
| 4 | FERMT2 |  |  | 227 | -0.259 | -0.3169 | No |
| 5 | SAT1 |  |  | 241 | -0.262 | -0.3238 | No |
| 6 | PPIB |  |  | 265 | -0.268 | -0.3482 | No |
| 7 | SERPINE1 |  |  | 323 | -0.286 | -0.4323 | No |
| 8 | MMP2 |  |  | 340 | -0.291 | -0.4427 | No |
| 9 | MEST |  |  | 352 | -0.299 | -0.4437 | Yes |
| 10 | COL4A2 |  |  | 353 | -0.300 | -0.4250 | Yes |
| 11 | ITGB5 |  |  | 356 | -0.300 | -0.4098 | Yes |
| 12 | LRP1 |  |  | 366 | -0.304 | -0.4069 | Yes |
| 13 | PMEPA1 |  |  | 375 | -0.308 | -0.4020 | Yes |
| 14 | RHOB |  |  | 391 | -0.316 | -0.4091 | Yes |
| 15 | TIMP1 |  |  | 399 | -0.320 | -0.4016 | Yes |
| 16 | GADD45A |  |  | 405 | -0.323 | -0.3904 | Yes |
| 17 | TPM1 |  |  | 407 | -0.327 | -0.3717 | Yes |
| 18 | CALD1 |  |  | 420 | -0.335 | -0.3723 | Yes |
| 19 | VEGFA |  |  | 436 | -0.345 | -0.3776 | Yes |
| 20 | SERPINE2 |  |  | 452 | -0.355 | -0.3823 | Yes |
| 21 | PDLIM4 |  |  | 466 | -0.370 | -0.3824 | Yes |
| 22 | ADAM12 |  |  | 472 | -0.374 | -0.3680 | Yes |
| 23 | NT5E |  |  | 500 | -0.416 | -0.3903 | Yes |
| 24 | JUN |  |  | 503 | -0.425 | -0.3673 | Yes |
| 25 | BDNF |  |  | 524 | -0.459 | -0.3744 | Yes |
| 26 | IGFBP3 |  |  | 538 | -0.493 | -0.3669 | Yes |
| 27 | COL4A1 |  |  | 545 | -0.507 | -0.3459 | Yes |
| 28 | TNFRSF12A |  |  | 550 | -0.519 | -0.3207 | Yes |
| 29 | GJA1 |  |  | 552 | -0.527 | -0.2895 | Yes |
| 30 | TIMP3 |  |  | 553 | -0.530 | -0.2564 | Yes |
| 31 | GPC1 |  |  | 555 | -0.536 | -0.2247 | Yes |
| 32 | TNC |  |  | 564 | -0.572 | -0.2033 | Yes |
| 33 | SPARC |  |  | 567 | -0.575 | -0.1710 | Yes |
| 34 | EMP3 |  |  | 568 | -0.580 | -0.1347 | Yes |
| 35 | CD44 |  |  | 575 | -0.659 | -0.1043 | Yes |
| 36 | SPP1 |  |  | 577 | -0.674 | -0.0640 | Yes |
| 37 | VIM |  |  | 578 | -0.674 | -0.0218 | Yes |
| 38 | PMP22 |  |  | 590 | -0.837 | 0.0107 | Yes |
Table: GSEA details [plain text format]

  

Fig 2: HALLMARK\_EPITHELIAL\_MESENCHYMAL\_TRANSITION: Random ES distribution      
 Gene set null distribution of ES for **HALLMARK\_EPITHELIAL\_MESENCHYMAL\_TRANSITION**
